# Effects of Arf6 downregulation on biological characteristics of human prostate cancer cells

**DOI:** 10.1590/S1677-5538.IBJU.2019.0499

**Published:** 2020-09-02

**Authors:** Haiming Lei, Fujun Ma, Renfeng Jia, Bo Tan

**Affiliations:** 1 School of Clinical Medicine Jiangsu Vocational College of Medicine YanchengJiangsu Province China School of Clinical Medicine, Jiangsu Vocational College of Medicine, Yancheng, Jiangsu Province, China;; 2 Department of Urology Shengli Oilfield Central Hospital DongyingShandong Province China Department of Urology, Shengli Oilfield Central Hospital, Dongying, Shandong Province, China

**Keywords:** Prostatic Neoplasms, Apoptosis, Neoplastic Stem Cells

## Abstract

**Objective:**

To evaluate the effects of Arf6 downregulation on human prostate cancer cells.

**Materials and Methods:**

The effects of Arf6 downregulation on cell proliferation, migration, invasion and apoptosis were assessed by MTT, BrdU, scratch, Transwell assays and flow cytometry respectively. AKT, p-AKT, ERK1/2, p-ERK1/2 and Rac1 protein expressions were detected by Western blot.

**Results:**

Downregulating Arf6 by siRNA interference suppressed the mRNA and protein expressions of Arf6. The proliferation capacities of siRNA group at 48h, 72h, and 96h were significantly lower than those of control group (P <0.05). The migration distance of siRNA group at 18h was significantly shorter than that of control group (P <0.01). The number of cells penetrating Transwell chamber membrane significantly decreased in siRNA group compared with that of control group (P <0.01). After 24h, negative control and normal control groups had similar apoptotic rates (P >0.05) which were both significantly lower than that of siRNA group (P <0.01). After Arf6 expression was downregulated, p-ERK1/2 and Rac1 protein expressions were significantly lower than those of control group (P <0.05).

**Conclusion:**

Downregulating Arf6 expression can inhibit the proliferation, migration and invasion of prostate cancer cells in vitro, which may be related to ERK1/2 phosphorylation and Rac1 downregulation.

## INTRODUCTION

Prostate cancer is one of the most common malignancies that endanger geriatric males. Its global incidence rate ranks second among those of all male malignancies. In 2012, there were approximately 1.1 million new cases of prostate cancer in the World ( 1 ). The occult onset of prostate cancer has no or unapparent symptoms in the early stage. Besides, tumor metastasis remains the main obstacle for prostate cancer treatment and the main cause of death ( 2 ).

As a member of the Ras gene superfamily, Arf can allosterically activate cholera toxin, enhance its ADP-ribosyl transferase activity, and promote its ADP-ribosylation on Gs protein α subunit. Arf6 is the most specific subtype of Arfs protein family with a molecular weight of about 20 kD, playing an important role in regulating endocytosis, extracellular secretion, endocytic membrane circulation, cell division, membrane lipid metabolism, microcapsule transport and release, formation of intercellular adhesion and junction, as well as cortical actin cytoskeletal remodeling ( 3 ). In addition, Arf6 is associated with membrane receptor endocytosis in clathrin-dependent and independent pathways ( 4 , 5 ). It is highly expressed in various tumor tissues and cells such as breast cancer ( 6 ), melanoma ( 7 ), glioma ( 8 ) and gastric cancer ( 9 ), and closely related to the invasion and metastasis of tumor cells.

The RNA interference technology can specifically and efficiently inhibit the expressions of specific genes in organisms. It has been widely used in gene functional studies, cellular signaling conductive pathways, drug target screening and clinical disease treatment. The aim of this study was to provide an experimental basis for revealing the roles of Arf6 gene in the proliferation, invasion, and metastasis of prostate cancer cells through RNA interference, and to design eligible therapies for metastatic prostate cancer using Arf6 as a target.

## MATERIALS AND METHODS

### Cell culture

Androgen-independent prostate cancer PC-3 and LNCap cell lines were purchased from American Type Culture Collection (USA). After resuscitation, the cells were cultured in RPMI-1640 medium containing 10% fetal bovine serum (FBS) and penicillin-streptomycin (100U/mL, Gibco, USA) in a 5% CO_2_ incubator at 37°C.

### Cell transfection

Arf6-specific small interfering RNA (siRNA) sequence was designed and synthesized by Guangzhou RiboBio Co.®, Ltd. (China) according to the Arf6 gene sequence in NCBI database (GenBank, NM-001663.3). Forward primer: 5’CUCACAUGGUUAACCUCUAdTdT3’, reverse primer: 5’UAGAGGUUAACCAUGUGAGTdTd3’. Meanwhile, nonsense sequence was synthesized as negative control. Forward primer: 5’CAACAAUCCUGUACAAGUUdTdT3’, reverse primer: 5’AACUUGUACAGGAUUGUUGdTdT3’.

Cells in the logarithmic growth phase were harvested and inoculated into 6-well culture plates. When the confluence reached 60-70%, the cells were transfected according to the siRNA instructions. Two sterile RNase-free microcentrifuge tubes were used. Each tube was added 200μL of Opti-MEM medium. One of the tubes was then added 7.5μL of Lipofectamine 2000, and the other was added 5μL of specific siRNA or NC-siRNA. The solutions in the two tubes were mixed evenly by a micropipette and left still for 15-20 min to form a complex. The siRNA/Lipofectamine 2000 complex (50nmol/L) was added into a 6-well plate, and the medium in each well was supplemented by fresh pre-warmed medium containing 10% FBS (without double antibody) to 2mL. The medium was refreshed for the first time 4-6h after transfection was completed. Forty-eight hours after transfection, the cells were digested by centrifugation with 0.05% trypsin, and collected to extract total RNA and total protein respectively. Reverse Transcriptase PCR (RT-PCR) and Western blot were used to detect the expression levels of target gene RNA and protein after siRNA interference, and to evaluate the efficiency of transfection.

## RT-PCR

RNA was quantified using NanoDrop2000 spectrophotometer (Thermo Fish Scientific, USA), and complementary DNA was synthesized with reverse transcriptase (Takara, Japan), RT-PCR was performed using SYBR-Green premix Ex Taq® (Takara, Japan) (n=3) and MxPro Mx3005P real-time PCR system® (Agilent, USA). RT-PCR primers were synthesized by Invitrogen (USA). ARf6, forward primer: 5’-ATGGGGAAGGTGCTATCCAAAATC-3’, reverse primer: 5’-GCAGTCCACTACGAAGATGAGACC-3’. GAPDH, forward primer: 5’-GGCCTCCAAGGAGTAAGACC-3’, reverse primer: 5’-AGGGGAGATTCAGTGTGGTG-3. Reaction conditions: Pre-denaturation at 95°C for 3 min, three-step amplification (40 cycles): 95°C for 20s, 60°C for 20s and 72°C for 20s. Using GAPDH as the internal reference, the relative expression of target gene was calculated by the 2-^CT^ method. Three replicate wells were set for each sample.

### Western blot

Total proteins were extracted by RIPA lysis buffer (Beyotime Institute of Biotechnology, Shanghai®, China) following the manufacturer’s protocol. Then 30mg proteins were separated by 10% sodium dodecyl sulfate-polyacrylamide gel electrophoresis and transferred onto a polyvinylidene difluoride membrane (Millipore®, USA). Afterwards, the membrane was blocked with 5% non-fat milk for 1h at 37°C, and incubated with anti-ERK1 (1:500, Cell Signaling Technology®, USA), anti-ERK2 (1:500, Cell Signaling Technology, USA), anti-Rac1 (1:800, Abcam, USA), anti-AKT (1:800, Abcam, USA), anti-p-AKT (1:800, Abcam, USA) and GAPDH (1:400, Santa Cruz Biotechnology, USA) antibodies for 1h at 37°C. After washing with tris-buffered saline containing 0.5% Tween 20 (TBST), the membrane was incubated with HRP-conjugated secondary antibody at 37°C for 40 min. After further washing with TBST, the membrane was color-developed with enhanced chemiluminescence reagent and exposed to X-ray film. The protein expression was quantified by Quantity One4.6.2 software, and the ratio of target protein to GAPDH protein level was calculated.

### MTT assay

The cell concentration of each group was adjusted to 1.0×10^3^, and then the cells were inoculated into a 96-well plate. Each well was added RPMI-1640 medium containing 10% FBS to 200μl, and the cells were cultured in 5% CO2 incubator at 37°C. Five replicates were set for each group. At indicated time intervals (0h, 12h, 24h, 48h, 72h and 96h after transfection), each well was added 20mL of MTT solution (5mg/mL), and the cells were cultured in 5% CO_2_ incubator at 37°C for 4h to generate formazan crystals. Subsequently, each well was added 150μl of dimethyl sulfoxide to dissolve the crystals. Finally, the optical density at 490nm was measured by a microplate reader.

### BrdU (5-Bromo-2’-deoxyuridine) assay

BrdU cell proliferation assay kit® (Biovision®, USA) was used for detection. The cells were seeded in a culture dish (diameter: 35mm) containing a coverslip at a density of 1.5×105/mL, cultured for 1 day and synchronized to the G0 phase in culture medium containing 0.4% FBS for 3 days. Before termination of culture, BrdU (final concentration: 30μg/L) was added, and the cells were incubated for 40 min at 37°C. The culture medium was discarded, and the coverslip was washed 3 times with PBS, fixed in methanol-acetic acid solution for 10 min and air-dried. After endogenous oxidase was inactivated by 0.3% H_2_O_2_ -methanol solution for 30 min, the coverslip was blocked with 5% normal rabbit serum, denatured by using formamide at 100°C for 5 min, cooled in an ice bath, washed with PBS and added anti-mouse BrdU monoclonal antibody (working concentration: 1:50). For negative control, PBS or serum was added. OD at 450nm was measured by the microplate reader.

### Scratch assay

Cells in the logarithmic growth phase were digested by 0.05% trypsin, centrifuged and resuspended in RPMI-1640 medium containing 10% FBS to prepare a single cell suspension at a density of 1.0×10^6^/mL. The cells were scratched with a 10μl micropipette tip. Three replicates were set for each group. After addition of RPMI-1640 medium containing 1% FBS, the cells were cultured in 5% CO_2_ incubator at 37°C. The migrating cells at different time points (0h and 18h) after scratching were photographed. When the scratches of the normal control group completely healed, the observation and photographing were ended. The results were statistically analyzed using Image J software.

### Transwell assay for in vitro migration and invasion

To monitor cell migration, the transfected cells were harvested, and 5×10^4^ cells in 200μL of medium containing 0.1% serum were placed in the upper chamber (pore size: 8μm) (BD Labware, USA®). The lower chamber was filled with medium containing 10% FBS (600μL). To monitor cell invasion, 5×10^4^ cells in 200μL of medium containing 0.1% serum were placed in the upper chamber which was precoated with Matrigel (BD Biosciences®, USA). After 24h of incubation, the cells were removed from the upper chamber using a cotton swab. The cells on the underside were fixed with 4% paraformaldehyde, stained with 0.1% crystal violet in 20% ethanol, and counted in five randomly selected visual fields under a phase contrast microscope. The migrating cells were photographed at 200× magnification under Leica microscope (Germany), in five independent fields per well. Each experiment was performed in triplicate.

### Flow cytometry

Cell apoptosis was analyzed by flow cytometry using annexin-V and propidium iodide (PI) double staining kit (Invitrogen®, USA) following the manufacturer’s protocol. Each experiment was performed in triplicate.

### Statistical Analysis

Each independent experiment was repeated three times. All data were analyzed by SPSS19.0 software and expressed as mean±standard deviation (x±SD). Pairwise comparisons were performed by the t test, and intergroup comparisons were conducted by one-way analysis of variance. P <0.05 was considered statistically significant.

## RESULTS

siRNA interference inhibited expressions of endogenous Arf6 mRNA and protein in prostate cancer cells.

**Figure 1:**
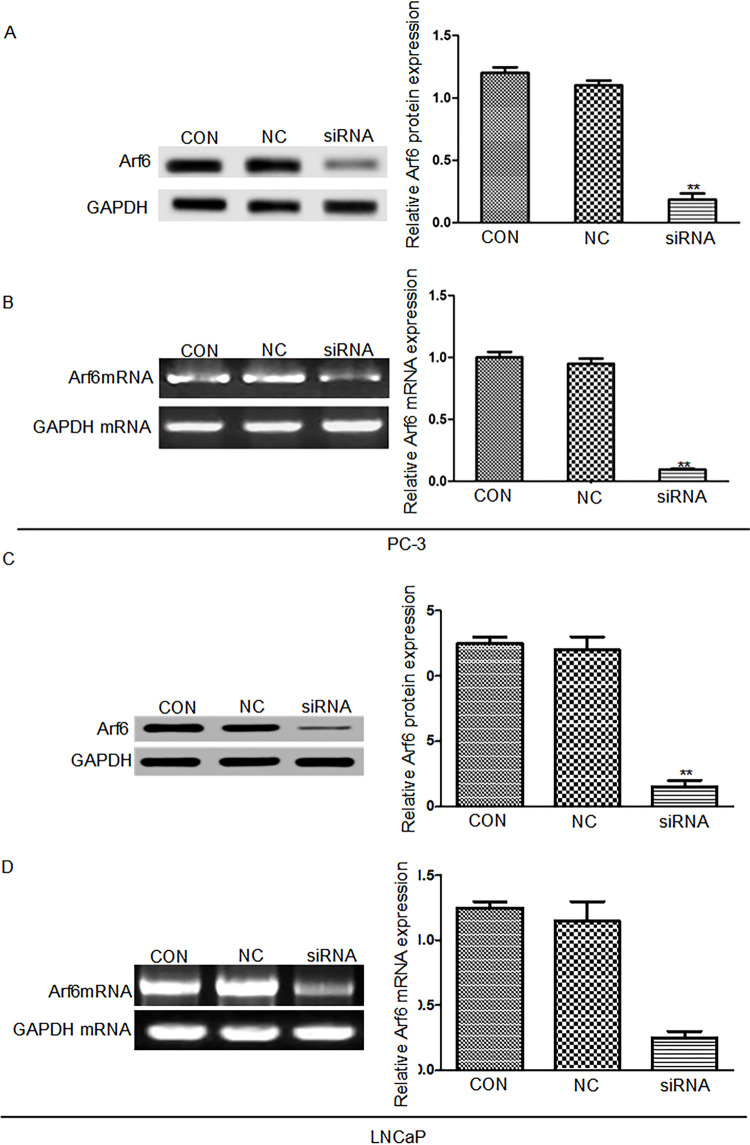
Arf6 expressions in prostate cancer cells after siRNA transfection. A and C: Arf6 protein expressions detected by Western blot, B and D: Arf6 mRNA expressions detected by RT-PCR. Con: Normal control group, NC: negative control group; siRNA: siRNA interference group. **Compared with Con and NC groups, P <0.01.

Forty-eight hours after transfection, the expression levels of Arf6 mRNA and protein in prostate cancer cells were detected by real-time PCR and Western blot respectively. The inhibition rates of Arf6 mRNA expression in PC-3 and LNCap cells were (91.88±3.13%) and (92.14±3.22%) respectively, and those of Arf6 protein expression in these two cell lines were (86.37±0.57)% and (85.79±0.64)% respectively ( Figure-1 ). In other words, after siRNA interference, the Arf6 mRNA expressions in PC-3 and LNCap cells decreased approximately 11.5-fold, and the protein expression reduced about 6.1-fold.

### Arf6 downregulation inhibited proliferation of prostate cancer cells

**Figure 2:**
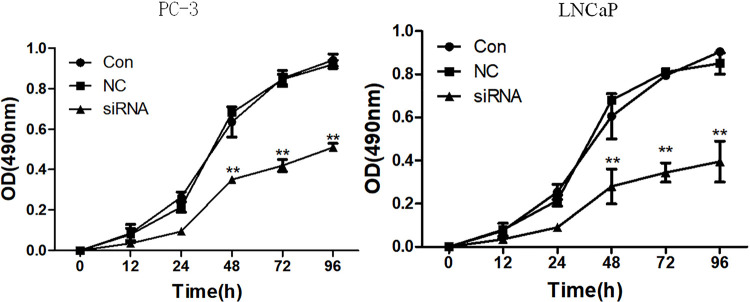
Effects of Arf6 downregulation on viability of prostate cancer cells evaluated by MTT assay. Con: Normal control group, NC: negative control group; siRNA: siRNA interference group. **Compared with Con and NC groups, P <0.01. **Compared with Con and NC groups, P <0.01.

The transfected prostate cancer cells were divided into siRNA group, negative control group and normal control group. The MTT assay showed that there was no significant difference in the viability between negative control and normal control groups (P >0.05), but the viabilities of the siRNA group were significantly lower than those of the control group at 48h, 72h and 96h (P <0.05) ( Figure-2 ).

**Figure 3:**
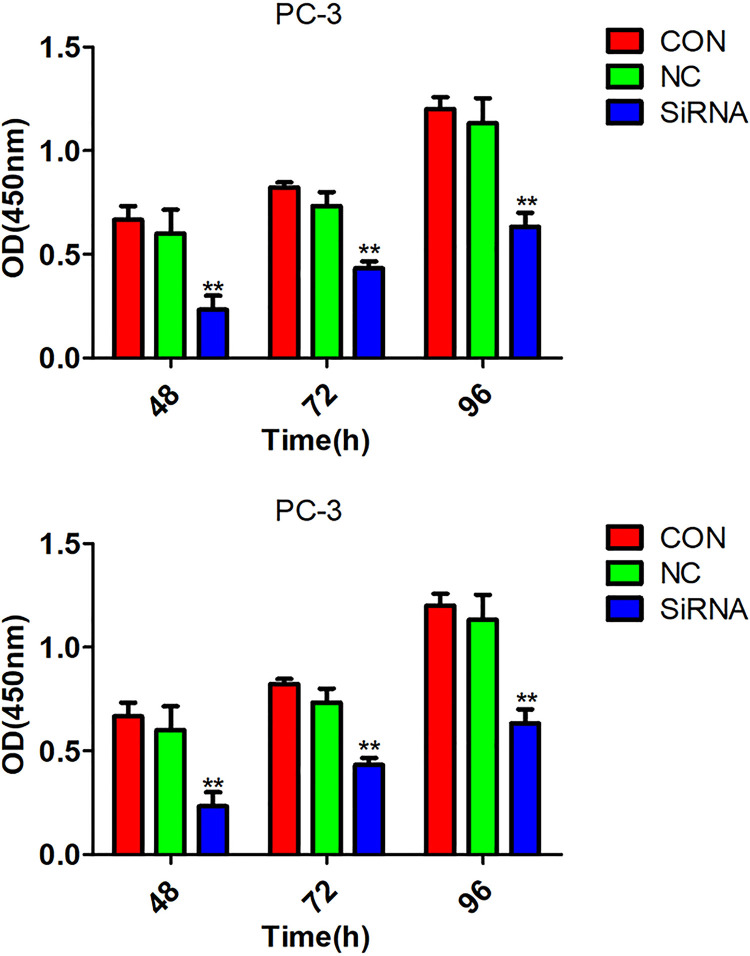
Effects of Arf6 downregulation on proliferative capacities of prostate cancer cells assessed by BrdU assay. Con: Normal control group, NC: negative control group; siRNA: siRNA interference group. **Compared with Con and NC groups, P <0.01.

The BrdU assay exhibited that the proliferative capacities of the siRNA group at 48h, 72h, and 96h were all significantly lower than those of the normal control group (P <0.05) ( Figure-3 ).

Arf6 downregulation inhibited migration and invasion of prostate cancer cells

**Figure 4:**
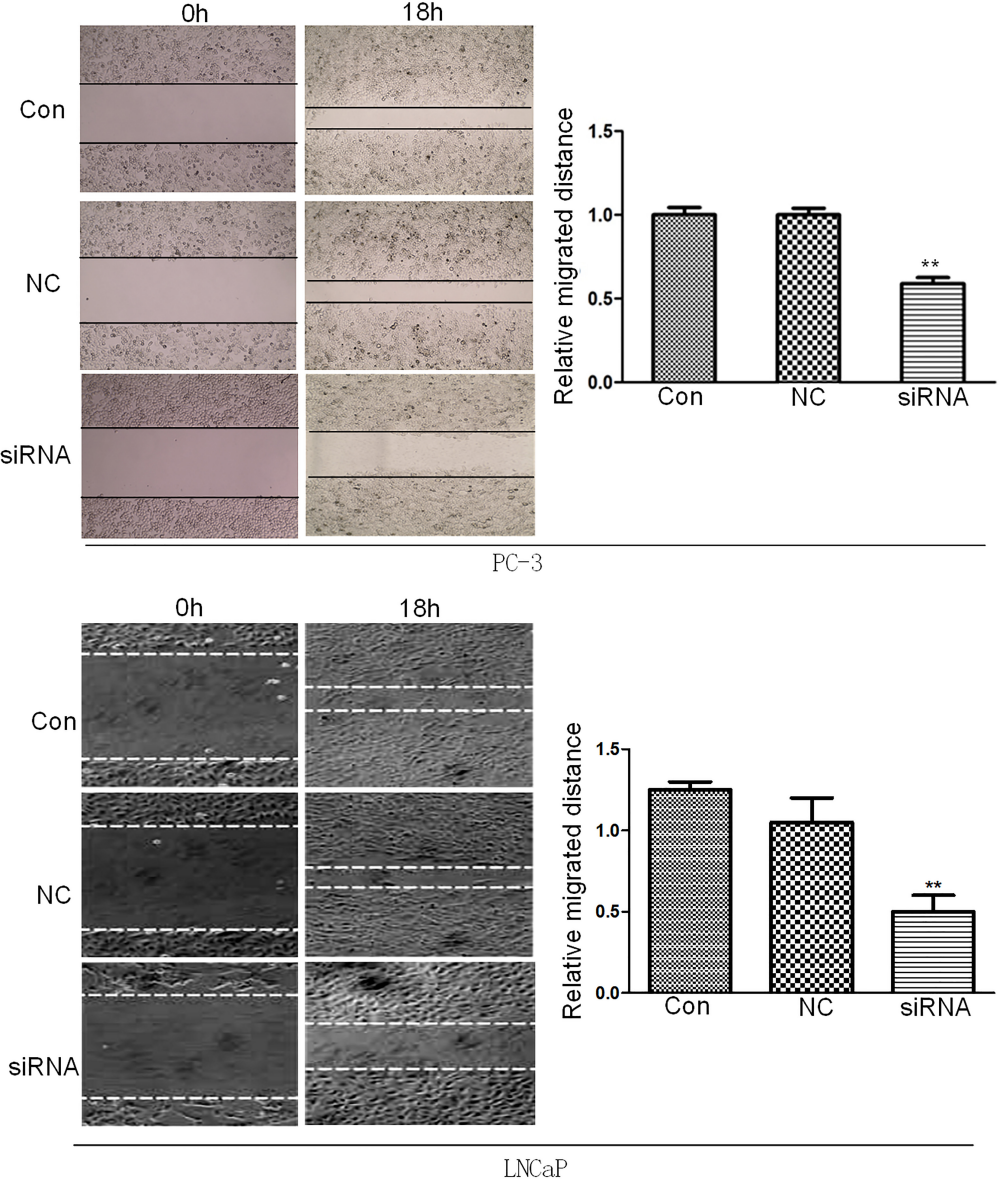
Effects of Arf6 downregulation on migration of prostate cancer cells tested by scratch assay. Con: Normal control group, NC: negative control group, siRNA: siRNA interference group. **Compared with Con and NC groups, P <0.01.

After the cells were scratched for 18h, there was no significant difference in the migration distance between negative control and normal control groups (P >0.05), whereas that of the siRNA group was significantly shorter than that of the control group (P <0.01) ( Figure-4 ).

**Figure 5:**
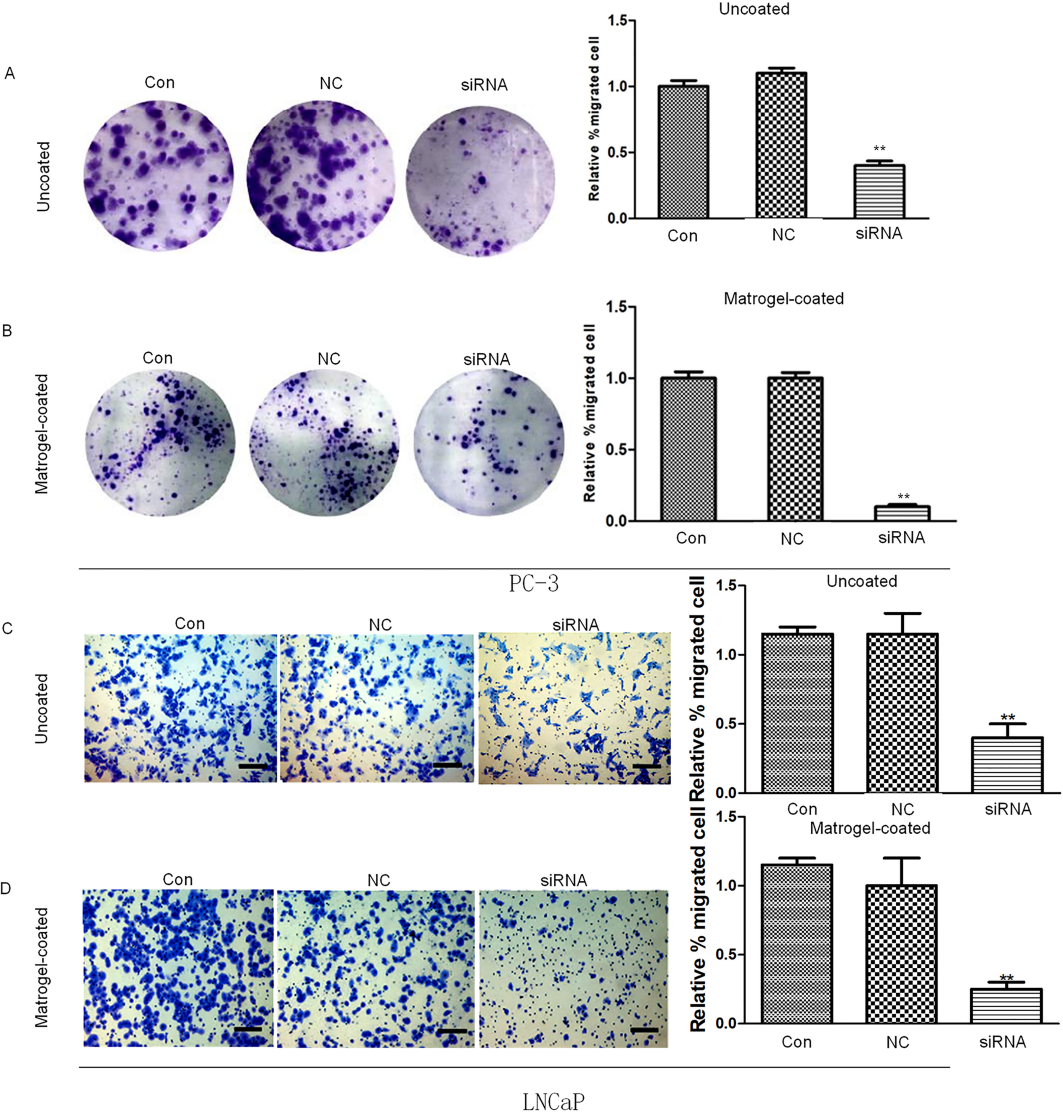
Effects of Arf6 downregulation on invasion of prostate cancer cells evaluated by Transwell assay. Con: Normal control group, NC: negative control group; siRNA: siRNA interference group. **Compared with Con and NC groups, P <0.01.

Additionally, the Transwell assay showed that the number of migrating cells in the negative control group was similar to that of the normal control group (P >0.05), but the number of the siRNA group was significantly lower than that of the control group (P <0.05) ( Figures 5A and C ). Therefore, downregulating Arf6 expression in prostate cancer cells effectively inhibited their migration. Besides, we used Matrigel with components similar to human extracellular matrix protein, and further coated Transwell chambers with Matrigel to assess the effects of downregulation of Arf6 expression in prostate cancer cells on their invasion. Figures 5B and D exhibit that the number of invasive cells in the siRNA group is significantly lower than that of the control group (P <0.05), but normal control and negative control groups have similar numbers (P >0.05). Collectively, downregulating Arf6 expression in prostate cancer cells effectively suppressed their migration and invasion.

### Arf6 downregulation promoted prostate cancer cell apoptosis

**Figure 6:**
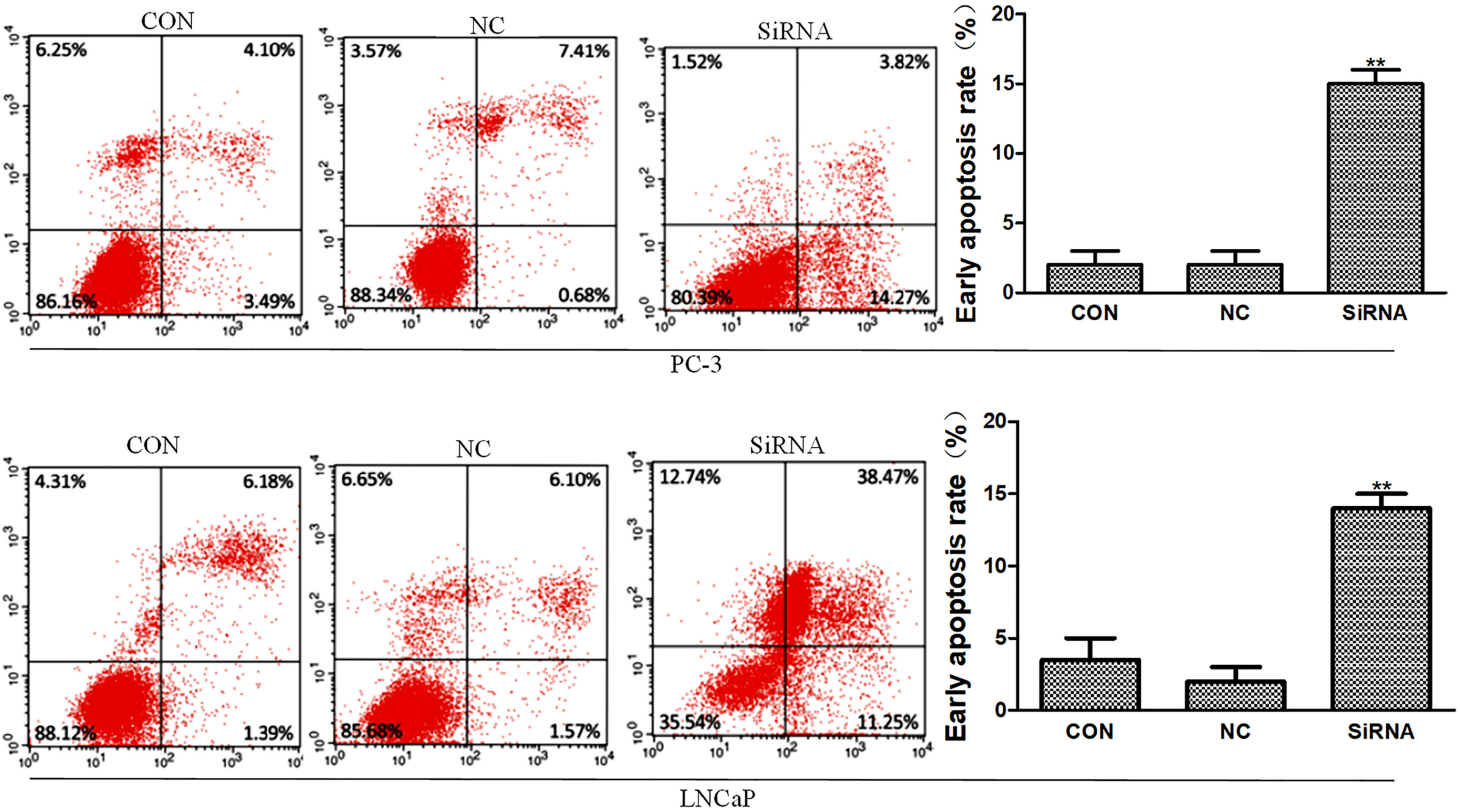
Flow cytometry showed that Arf6 downregulation promoted prostate cancer cell apoptosis. Con: Normal control group, NC: negative control group, siRNA: siRNA interference group. **Compared with Con and NC groups, P <0.01.

The apoptosis of prostate cancer cells was detected by annexin-V and PI double staining. After 24h, the negative control and normal control groups had similar apoptotic rates (P >0.05), which were both significantly lower than that of the siRNA group (P <0.01) ( Figure-6 ).

### Arf6 downregulation inhibited expressions of p-ERK1/2 and Rac1 but not AKT activity

**Figure 7:**
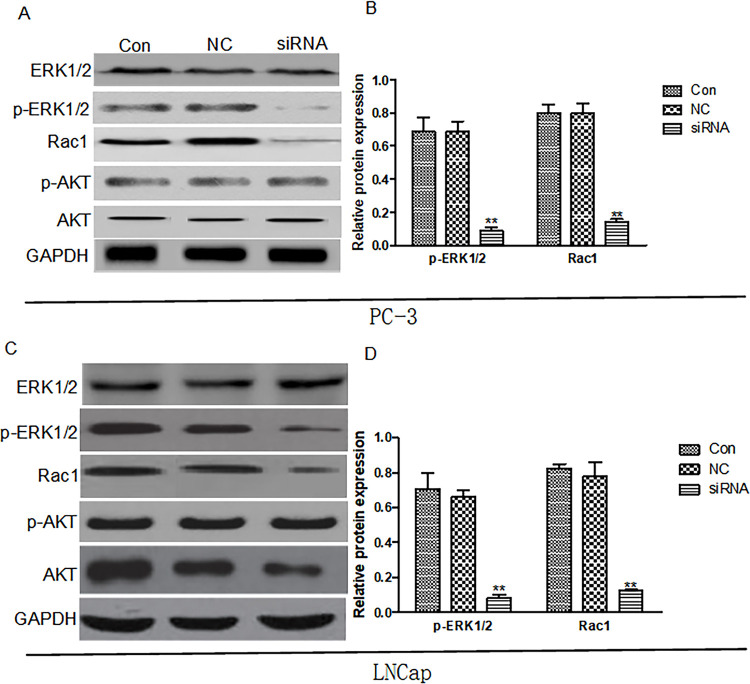
Expressions of key signaling molecules in PI3K/AKT and ERK/Rac1 pathways detected by Western blot. Con: Normal control group, NC: negative control group, siRNA: siRNA interference group. **Compared with Con and NC groups, P <0.01.

Increase of Arf6 expression can promote glioma cell proliferation, which is associated with the activation of PI3K/AKT signaling pathway ( 10 ). To further explore whether siRNA can interference with Arf6 expression to inhibit the proliferation of prostate cancer cells via the PI3K/AKT signaling pathway, Western blot was conducted to detect the expression and phosphorylation of AKT, a key molecule in this pathway. As shown in Figures 7A and C , the expression levels of AKT and p-AKT proteins in negative control, normal control and siRNA groups are not significantly different, suggesting that Arf6 downregulation suppressed the proliferation of prostate cancer cells not via the PI3K/AKT signaling pathway. Arf6 can regulate tumor cell proliferation, migration and invasion via the ERK signaling pathway ( 11 ). Accordingly, we used the same method to detect the expressions of ERK1/2 and p-ERK1/2. There was no significant difference in the expression of ERK1/2 protein among negative control, normal control and siRNA groups, but the expression of p-ERK1/2 in the siRNA group was significantly lower than that in the control group, indicating that inhibition of the proliferation, invasion and migration of prostate cancer cells through downregulating Arf6 expression may be related to the downregulation of p-ERK expression. Moreover, downregulating Rac1 expression can significantly inhibit the proliferation, invasion and metastasis of prostate cancer cells ( 12 ). To study whether the inhibitory effects of Arf6 downregulation on the proliferation, invasion, and migration of prostate cancer cells were related to Rac1, we detected Rac1 protein expression. As presented in Figure 7B and D , the Rac1 protein expression of the siRNA group is significantly lower than that of the control group.

## DISCUSSION

Arf6, which is located in mitotic intermediates, can participate in the regulation of mitotic activity as well as tumor cell growth and proliferation ( 13 ). Knizhnik et al. found in HET SR cells that overexpression of wild-type Arf6 or continuous activation of Arf6 mutant significantly enhanced cell proliferation ( 14 ). Koumakpayi et al. reported that Arf6 regulated the nuclear localization of ErbB3, a member of the EGFR family, in prostate cancer cells ( 15 ).

To explore the role of Arf6 in prostate cancer cell proliferation, we used siRNA to downregulate endogenous Arf6 expression in androgen-independent prostate cancer cells. The proliferative capacity after Arf6 downregulation was significantly lower than that of the control group, suggesting that Arf6 played a key role in the proliferation of prostate cancer cells. Also, Arf6 can promote the change of tumor cell morphology and the formation of invasion-related membrane synapse structure, degrade extracellular matrix proteins, enhance tumor cell movement, migration and invasion, and promote distant metastasis mainly through regulating tumor cell endosmosis cycle, troponin skeleton remodeling and release of microvesicles ( 16 ). In this stud, downregulation of Arf6 expression by siRNA interference inhibited prostate cancer cell movement, migration and invasion. Consistently, Hu et al. found that siRNA interfered with the expressions of endogenous Arf6 in breast and hepatoma cells and inhibited their invasion and migration ( 17 ).

Activation of the PI3K/AKT signaling pathway can promote prostate cancer cell growth and proliferation ( 18 ). Herein, we detected the expressions of AKT and p-AKT by Western blot and found that they were similar among different groups. Therefore, Arf6 regulated prostate cancer cell proliferation probably not via the PI3 K/AKT signaling pathway. Additionally, the regulatory effects of Arf6 on cell proliferation have been related with the ERK1/2 activity ( 19 ). In this study, Arf6 downregulation significantly inhibited the expression of p-ERK 1/2 in PC-3 cells, suggesting that such downregulation may be associated with decreased expression of p-ERK1/2. In addition, Arf6 has also been reported to regulate tumor cell invasion and migration via the ERK signaling pathway, and to promote melanoma cell invasion and metastasis by enhancing ERK1/2 phosphorylation ( 7 ). In this study, after Arf6 expression was downregulated, the phosphorylation of ERK1/2 in prostate cancer cells was significantly suppressed. Hence, downregulating Arf6 modulated the migration and invasion of prostate cancer cells possibly through the downregulation of p-ERK1/2.

Rac1 is a member of the Rho family of small G proteins that can regulate actin cytoskeleton remodeling and promote the formation of cell membrane synaptic structures linked to tumor cell invasion and migration ( 20 ). Downregulating Rac1 expression by siRNA or protein inhibitors can reduce the formation of dendritic pseudopods on the surface of cell membrane by inhibiting the skeleton remodeling of prostate cancer cells and suppress their metastasis ( 21 ). Hu et al. found that Arf6 enhanced the Rac1 activity by recruiting IQGAP 1 to facilitate glioma cell invasion and migration ( 17 ). However, it was found in hepatoma cells that downregulating Arf6 expression suppressed the activity of its downstream effector Rac1 by attenuating the phosphorylation level of ERK1/2, which reduced the invasion and migration of hepatoma cells ( 22 ). We herein found that Rac1 protein expression significantly decreased after Arf6 expression was downregulated. The inhibitory effects of Arf6 downregulation on the migration and invasion of prostate cancer cells may be related to the decrease of Rac1 expression. In addition, Kobayashi et al. reported that Rac1 downregulation induced the arrest of prostate cancer cell cycle in the G1/S phase and inhibited cell proliferation ( 23 ). In this study, after Arf6 was downregulated, both the cell proliferative capacity and Rac1 expression reduced, indicating that the molecular mechanism by which prostate cancer cell proliferation was suppressed by Arf6 downregulation may be the downregulation of Rac1 expression. Notably, Knizhnik et al. reported that Arf6 promoted tumor cell proliferation via the PLD-mTORC1 and p38MAPK pathways ( 14 ). Hence, in future studies, we will detect the expression changes of key proteins in these pathways and add specific blockers to clarify their roles in prostate cancer.

## CONCLUSIONS

In summary, downregulating endogenous Arf6 expression in androgen-independent prostate cancer cells by siRNA interference can significantly reduce their proliferation, migration and invasion. The molecular mechanism may be related to the downregulation of p-ERK1/2 and Rac1 expressions.
